# The possible link between Fetuin-A Protein and Neuro-inflammation in Children with Autism Spectrum Disorder

**DOI:** 10.12669/pjms.37.4.4032

**Published:** 2021

**Authors:** Laila Yousif Al-Ayadhi, Farah Ali Alghamdi, Lamees Abdula Altamimi, Luluh Yousef Alsughayer, Abdulrahman Mohammed Alhowikan, Dost Muhammad Halepoto

**Affiliations:** 1 Laila Yousif Al-Ayadhi, PhD. Autism Research and Treatment center, Department of Physiology, Faculty of Medicine. King Saud University, P.O. Box: 2925, Riyadh 11461, Saudi Arabia; 2 Farah Ali Alghamdi, MBBS. Faculty of Medicine, Dar Al Uloom University, Al Falah, Riyadh 13314, Saudi Arabia; 3 Lamees Abdula Altamimi, MBBS. College of Medicine, King Saud University, P.O. Box: 2925, Riyadh 11461, Saudi Arabia; 4 Luluh Yousef Alsughayer, MBBS College of Medicine, King Saud University, P.O. Box: 2925, Riyadh 11461, Saudi Arabia; 5 Abdulrahman Mohammad Alhowikan, PhD. Department of Physiology, Faculty of Medicine, King Saud University, P.O. Box: 2925, Riyadh 11461, Saudi Arabia; 6 Dost Muhammad Halepoto, PhD. Autism Research and Treatment Center (99), Department of physiology, Faculty of Medicine, King Saud University, P.O. Box: 2925, Riyadh 11461, Saudi Arabia

**Keywords:** Fetuin-A, Neuro-inflammation, Autism Spectrum Disorder

## Abstract

**Objectives:**

To investigate the blood plasma levels of Fetuin-A protein in children with Autism Spectrum Disorder (ASD) and healthy controls that could offer novel diagnostic biomarkers of disease development in ASD. Another objective was to investigate the severity of autistic children by Childhood Autism Rating Scale (CARS) and Short Sensory Profile (SSP).

**Methods:**

This case control study was carried out at Autism Research and Treatment (ART) Center, King Saud University, Riyadh, Saudi Arabia, from October 2019 to February 2020. Plasma concentration of Fetuin-A was analyzed by enzyme-linked immunosorbent assay (ELISA) in ASD subjects (n=46) and normal controls (n=44). Correlation among Fetuin-A levels, CARS and SSP was established by Spearman’s correlation coefficient (r).

**Results:**

Overall, autistic children had significantly (p= 0.0.02) lower Fetuin-A concentration [50.76 (22.2-68.5) ng/ml] than those of healthy controls [53.7 (35.6-99.7) ng/ml] [median (interquartile range)]. Children with mild to moderate autism (n=24, 52%) also showed significantly lower Fetuin-A levels [50.0 (30.0-68.2) ng/ml], (p =0.02} than healthy controls [53.7 (35.6-99.7) ng/ml] [median (IQR)]. However, there was no significant change (p = 0.71) observed between the Fetuin-A levels of children with severe autism [51.8 (22.2-68.5)] ng/ml, mild to moderate autism [50 (30-68.2)] ng/ml [median (IQR)] and healthy controls (p=0.12). Also no significant correlations between Fetuin-A, CARS and SSP were observed (CARS, r= 0.024, p=0.88; SSP, r= -0.003, p=0.98).

**Conclusion:**

Overall the low Fetuin-A plasma values in ASD subjects, most likely show that Fetuin-A could be associated in the physiology of autism. Further studies with larger patient and control cohorts will be necessary to determine whether Fetuin-A can be used as a biomarker for ASD.

## INTRODUCTION

“Autism is a complex neurodevelopmental disorder characterized by impairments in social interaction, abnormalities in verbal and nonverbal communication and, restricted, stereotyped interests and behaviors”.^1^ In spite of intense research efforts over the past several years, the pathophysiology of ASD is still not known, and there are no specific biological or clinical markers for diagnosing ASD. Additionally, to explore possible biomarkers, numerous research studies have investigated proteome characteristics in the blood of autistic subjects.^2^ The neurological origin for ASD is still not well known, and the link between neuro-inflammation and autoimmunity requires to be explored. This work emphasizes the role of the Fetuin-A Protein in neuro-inflammation linked to autoimmunity and to examine the link between Futine –A and severity of autism.

Serum glycoprotein Fetuin-A is the member of the cystatin protein group, mostly formed in the liver. The Fetuin-A protein is necessary for mineral homeostasis and demonstrates immunomodulatory roles, for instance to bind with TGF super family proteins.^3^ Fetuin-A has been revealed to have anti-inflammatory functions and it has neuroprotective effect in Alzheimer’s disease (AD).^4^ Reduced Fetuin-A Protein is an indicator of amplified disease action in ulcerative colitis, Crohn’s disease and obstructive lung disease.^4^ However, the pathophysiological role of Fetuin A in ASD is uncertain. It is involved in several biological functions, such as, formation of bone tissue, endocytosis and the brain development.^3^ Fetuin-A protein is usually found in brain tissues and mostly in fetal organs. Fetuin-A is neuroprotective in animal models of lethal chronic inflammation and cerebral ischemia signifying a role beyond the neonatal age.^2^ However, the occurrence of Fetuin-A in the brain tissue of adult human under dissimilar pathological and physiological states is little known.^5^

Immunological irregularities have been associated to ASD and these deformities are produced by brain inflammation.^6^ Vargas et al^7^ have reported increased neuroinflammation in subjects with ASD. Fetuin-A anticipates vascular threat in a diversity of clinical settings and it shows to be anti-inflammatory in cerebral ischemia. Assumed the obvious significance of neuroinflammation in cognitive decrease Smith et al^8^ examined Fetuin-A and pro-inflammatory cytokine levels in AD patients, and examined the association among Fetuin-A levels and a cognitive impairment. Shi et al^9^ explored the expression change of Fetuin-A protein as anti-inflammatory marker in mice brain of a novel AD model and reported a lower Fetuin-A protein expressions in the, hippocampus, thalamus and cerebral cortex in a mice brain.

At present there is crucial necessity for biomarkers in ASD that can authenticaly determine continuous disease progress in relation to autoimmunity and inflammation. Fetuin-A has been recognized as a biomarker for neurodegenerative disease^10^ and shows protective outcomes in inflammation.^11^ Recently Fetuin-A was also recognized as a possible biomarker in multiple sclerosis (MS) cerebrospinal fluid (CSF) of mice.^12^ Puchades et al^13^ compared the CSF proteome between controls and AD patients and identified that Fetuin-A is altered and contributed in the disease process and pathogenesis of AD. Protein analysis for possible biomarkers in blood plasma of mild AD patients and normal controls was also carried out and three down regulated proteins including Fetuin-A were identified.^14^

Fetuin-A was linked with severity of disease in autoimmune inflammation.^12^ Fetuin-A Protein CSF levels were considerably greater in active MS patients and decreased with complete treatment.^12^ Fetuin-A concentration was low in patients with AD in ratio to the severity of cognitive loss. Laughlin et al^15^ assessed the relationship of Fetuin-A serum concentrations with cognitive role in community-home elderly people and concluded that increased Fetuin-A levels are linked with improved performance on tests of worldwide cognitive and administrative role with less possibility of main decrease in these cognitive skills with duration of four year. Recently Fanelli et al suggested lower Fetuin-A level as potential biomarker of depression in elderly people.^16^

Oxidative stress plays a key role in the pathogenesis of several chronic and acute neurological disorders including ASD.^17^ Kanno et al^18^ proposed that Fetuin- A is strongly associated to the cytoprotective activity against oxidative injury in neuronal cells in vitro. Recent Proteome study^1^ discovered eighty two (82) altered proteins including Fetuin- A in the subgroup of ASD with severe language difficulties. Brettschneider et al^19^ recognized Fetuin-A protein that was changed in CSF of amyotrophic lateral sclerosis (ALS) patients with a fast development of disease as observed by compromised motor function.

The basic pathogenic mechanism in autoimmune disorders, is the creation of antigen-antibody complexes which activate an inflammatory response by inducing the infiltration of neutrophils. We hypothesized that Fetuin-A, could play a pathogenic role in inflammation and autoimmune disorders. These findings direct us to explore for biological markers that may permit prior discovery of autism. Since plasma levels of Fetuin-A, in patients with ASD have never been reported previously, therefore, the main object of present study was to discover a promising role for Fetuin-A, in children with ASD and investigate the severity of diseases by CARS and SSP scores.

## METHODS

### Participants

This case control study was carried out at Autism Research and Treatment (ART) Center, King Saud University, Riyadh, Saudi Arabia, from October 2019 to February 2020. Forty-six (46) male children with autism, aged 3-13 years (mean ± SD = 5.9 ± 2.4 years), from the Autism Research and Treatment (ART) Center at King Saud University Riyadh, Saudi Arabia and the control group consisted of 44 age and sex-matched healthy children from pediatric clinic at the King Saud medical city Riyadh, Saudi Arabia were enrolled in this study. ASD was diagnosed according to the Diagnostic and Statistical Manual of Mental Disorders (DSM-5).^1^ Participants associated with obsessive-compulsive disorder, epileptic seizures, affective disorders, fragile X syndrome, or any additional psychiatric or neurological diseases were excluded from the study. This study was approved by the IBR Committee of the King Khalid Hospital at King Saud University in Riyadh, Saudi Arabia. Informed written consent was signed by the parents or the legal guardians of all the enrolled participants. Autism severity was measured in all children with ASD by using CARS and SSP scales as described elsewhere.^20^

### Blood sample collection

“Blood samples from 46 ASD and 42 matched healthy children were drawn after overnight fasting. Blood was taken into 3 mL EDTA containing tubes for blood collection. The samples were centrifuged directly after the blood sampling for 20 min at 4 °C at 3000×g. Until analysis, the plasma was kept at −80 °C. Fetuin-A concentrations were measured in the plasma of all subjects using a commercially available sandwich ELISA kit (Cusabio Biotech Co. Ltd., Wuhan, China).”

### Statistical analysis

“All results were analyzed using a commercially available software package, IBM SPSS Statistics Version 21. Age and Fetuin-A are presented with median and IQ range. Because of non-normally distributed continuous variables, the Mann-Whitney U test was used for comparisons of Fetuin - A, protein levels between the autistic and control groups. The null hypothesis was that there would be no difference in the data distributions for Fetuin - A, protein levels between participants diagnosed with ASD and control children. The Spearman’s rank-correlation coefficient test (r) was used to test for correlation between Fetuin - A and different variables (CARS and SSP). For all statistical tests employed in the present study, a two-tailed p-value ≤ 0.05 was considered statistically significant.”

### Ethical Committee Approval

(Ref: 20/0741/IRB, Dated: 15-10-2020).

## RESULTS

The general features of the study subjects and plasma levels of Fetuin-A in autistic children (n=46) and healthy controls (n=44) are shown in Table-1. Levels of Fetuin-A were compared between children with different severity of autism (severe or mild-moderate) and age-matched healthy controls. Severity of ASD in children was classified according to their recorded CARS score, 20 which ranged from 30 to 65. The sensory functions of autistic children were recorded by SSP score^20^.

**Table-I:** General characteristics and plasma levels of Fetuin-A protein in ASD children and controls and their association with Autism severity.

Group	Age (Years) Median (IQ range)	Fetuin-A (ng/ml) Median (IQ range)	p-value	CARS score	SSP score
Children with Autism (n=46)	5 (3-13)	50.76 (22.2-68.5)	0.02*	≥ 30	
Children with severe Autism (n=22)	5(3-13)	51.8 (22.2-68.5)	0.12** 0.71****	>36.5	< 142
Children with mild to moderate autism (n=24)	4.5 (3-12)	50 (30-68.2)	0.02***	<36.5	>142
Healthy children/ Control (n=44)	6 (3-13)	53.7(35.6-99.7)			

* Comparing autistic children with control subjects;

** Comparing severe autistic children with control subjects;

*** Comparing mild to moderate autistic children with control subjects;

**** Comparing mild to moderate autism with severe autistic children. (p-value ≤ 0.05 was considered statistically significant).

Overall, Fetuin-A level of autistic children (n = 46 and CARS score >30) exhibited significantly lower (p= 0.02) plasma level [50.76 (22.2-68.5)] ng/ml [median (interquartile range)] than those of healthy controls [53.7(35.6-99.7)] ng/ml [median (IQR)].There was also a significant (p=0.02) difference observed between the Fetuin-A level [50 (30-68.2)] ng/ml [median (IQR)] of mild to moderate autism (n = 24 and CARS score <36.5) and healthy controls [53.7(35.6-99.7)] ng/ml. However, there was no significant difference (p = 0.71) was observed between the Fetuin-A levels [51.8 (22.2-68.5) of children with severe autism (n = 22 and CARS score >36.5), mild to moderate autism [50 (30-68.2)] ng/ml [median (IQR)] and healthy controls (p=0.12).

Autistic children also showed certain level of sensory processing dysfunction on the SSP total score. Children with severe and moderate autism show 141± 26.3 and 148± 25.4 mean SSP scores respectively which are associated with severe and mild to moderate presentation (clearly different from a typical presentation).

**Fig.1:**
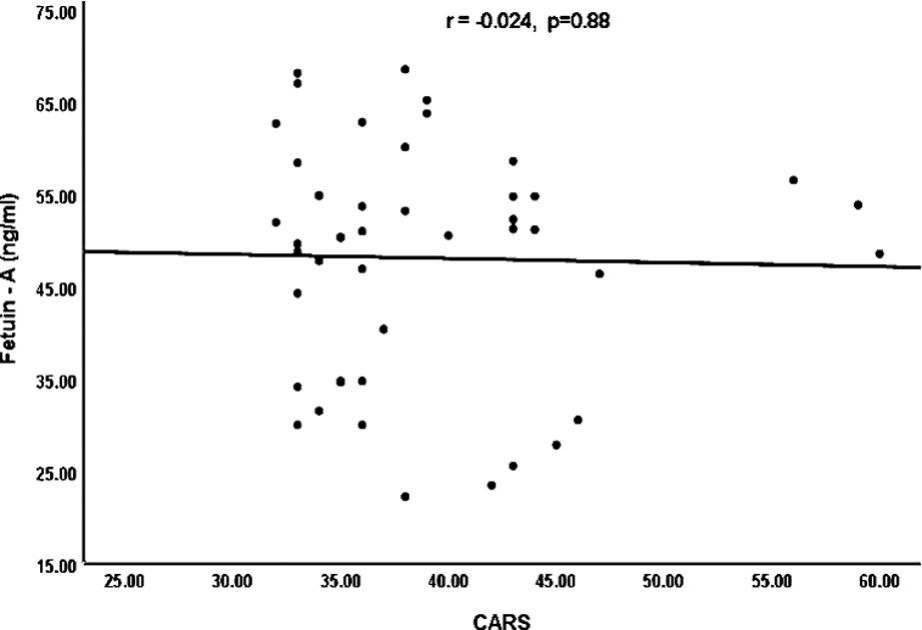
Correlation between Fetuin - A protein and childhood autism rating scale (CARS).

**Fig.2:**
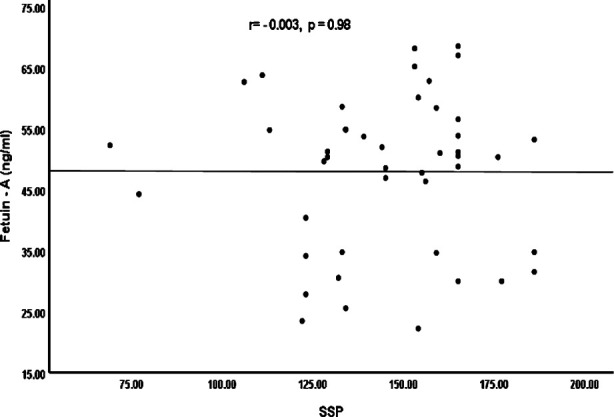
Correlation between Fetuin - A protein and short sensory profile scale (SSP).

Spearman’s correlation coefficient (r) was calculated to determine the relationships between Fetuin-A levels and different variables (CARS, SSP) as shown in Figures 1 and 2. However, resulting graphs showed no significant correlations between Fetuin-A levels and these variable scales (CARS, r= -0.024, p=0.88; SSP, r= -0.003, p=0.98).

## DISCUSSION

The etiopathogenesis of ASD is not well known, however it appears to involve dysfunction in many biological systems. Biomarker innovation research in ASD has been mainly limited to peripheral tissue, such as blood. Blood based biomarkers can be important in the progress of analytical tools to identify disease or helpful as companion diagnostics to classify patients most likely to benefit from specific pharmacotherapies.^21^

This study investigated Fetuin-A, as biomarker for the early diagnosis of autism. Previous research showed that Fetuin-A, is linked with neuroinflammation in subjects with ALS and MS.^12^,^19^ Tentatively the dysregulation of Fetuin-A in ASD patients might be linked with inflammatory mechanisms related with microglial activation in ASD.

To best of our knowledge, this is the first study to assess plasma Fetuin-A, levels and its association with CARS and SSP in autistic children. Fetuin-A, levels were found significantly lower in children with ASD compared with the control group. We could not find any single study in updated research about the role of Fetuin-A protein in ASD children to match our data. However, one study using multi-omics analyses revealed that reduced levels of diazepam-binding inhibitor (DBI) proteins including Fetuin-A were associated with severe language impairment in ASD.^2^ Furthermore, our findings were also consistent with earlier studies showed decreased levels of Fetuin-A in Alzheimer’s disease (ALD), major depressive disorder (MDD).^8^, ^16^ Though caution should be applied in the interpretation of our rdata, suggesting a possible role for Fetuin-A in ASD.

Fetuin-A plays key role for the brain development.^2^ Our findings are consistent with the hypothesis that decreased Fetuin-A may result in the compromised anti-infammatory activity in brain of ASD, where mechanism depends on the accessibility of Fetuin-A with increased microglial activation.^22^ At higher concentrations as in healthy controls Fetuin-A acts as an anti-inflammatory, and efficiently reduces bacterial endotoxin induced formation of proinflammatory mediators (such as TNF, IL-1, and nitric oxide) in macrophage cultures.^23^

As the classification of ASD as a neurological disease, sensory impairment is currently believed an autistic characteristic^24^. It is presently reported that 45–96% children with ASD have sensory dyfunction. The behaviors of children with ASD are associated to difficulties with sensory reflections, which result social deficits for these children and restrict their everyday life activities.^25^ Search of different biomarkers to determine the characteristics of autism severity, like sensory defects and cognitive dysfunction, can provide us a enhanced knowledge of the ASD pathophysiology. The current study also explored the correlation of Fetuin-A levels with CARS and SSP among children with ASD. However, plasma Fetuin-A levels were not significantly correlated with CARS (r=−0.024, p=0.88) and SSP (r=-0.003, p=0.94) scores among the ASD patients, resulting Fetuin-A may not be associated to the severity or development of disease.

We may conclude that reduced Fetuin-A levels in children with ASD might be clarified, due to the occurrence of inflammation in CNS. Potentially significant role for Fetuin-A in controlling inflammatory responses is under current research. The fundamental biology of Fetuin-A is complicate, and as with various Fetuin-A roles, based on the biological background. Therefore, mechanisms excluding usual pathogenic pathways are probably to be implicated in the biology underlying the Fetuin-A association. This study also provides information that higher Fetuin-A may also offer immunity in normal controls as compared to ASD subjects. It also supports the potential association between low Fetuin-A levels and neurodevelopmental changes in ASD.

The initial results obtained in present study look promising; and on the basis of the preliminary results, it appears to be evocative indication in support of Fetuin-A involvements to the pathophysiology of autism. Further studies aimed to develop on these results are warranted. Known the key role of Fetuin-A in neuroinflamation in other neuro diseases^12^, ^19^ the present findings also lead to the assumption that lower levels of Fetuin-A may be responsible for the neuroinflamation caused by autoimmunity in ASD. This study should be considered as a preliminary study to discover Fetuin-A as potential biomarker of disease progression in ASD. Additional research on larger population is required to prove the significance of the Fetuin-A and establish diagnostic accuracy to recognize ASD patients at risk of a fast advancement of disease.

### Limitations of the Study

It is cross-sectional design and small sample size. Therefore, association was observed not causation or prediction. Additional limitation of this study is that adaptive behavior was not evaluated in the control group. Thus, whether Fetuin-A levels may be linked with behavioral characteristics in healthy controls remains to be clarified. These results offer preliminary, direct evidence of altered Fetuin-A protein in subjects with ASD, which may contribute to the early pathogenesis of ASD, provide valuable biomarker, and lead to novel therapeutic interventions.

## CONCLUSION

Fetuin-A levels were found to be significantly lower in children with ASD compared to normal healthy children however they were not significantly associated with the CARS and SSP. Our findings suggested that Fetuin-A may play a role in the neuroinflammationin in autism and this protein might act as candidate protein for upcoming research into the ASD mechanisms at molecular level or discovery of ASD biomarkers. Studies with larger samples are necessary to increase our knowledge about the role of Futine-A in ASD. However, these results should be treated with caution till additional studies are carried out on larger scale to decide whether the reduction of Fetuin-A level is a mere consequence of autism or has a pathogenic role in the disease.

### Author`s Contribution:

**LYA:** Conceived the idea and designed the study. Approval of the final version to be published.

**FAG:** Data collection and manuscript writing.

**LAA:** Data analysis and data interpretation.

**LYA:** Data analysis and data interpretation.

**AMA:** Drafting and revising the paper.

**DMH:** Literature search and final review, also the responsible for the accuracy work.

## References

[1] American Psychiatric Association Diagnostic and statistical manual of mental disorders (2013). American Psychiatric Association, Washington.

[2] Pichitpunpong C, Thongkorn S, Kanlayaprasit S, Yuwattana W, Plaingam W, Sangsuthum S (2019). Phenotypic subgrouping and multi-omics analyses reveal reduced diazepam-binding inhibitor (DBI) protein levels in autism spectrum disorder with severe language impairment. PLoS One.

[3] Elsas J, Sellhaus B, Herrmann M, Kinkeldey A, Weis J, Jahnen-Dechent W (2013). Fetuin-A in the developing brain. Dev Neurobiol.

[4] Mukhopadhyay S, Mondal SA, Kumar M, Dutta D (2014). Proinflammatory and antiinflammatory attributes of fetuin-a:a novel hepatokine modulating cardiovascular and glycemic outcomes in metabolic syndrome. Endocr Pract.

[5] Heinen MC, Babler A, Weis J, Elsas J, Nolte K, Kipp M (2018). Fetuin-A protein distribution in mature inflamed and ischemic brain tissue. PLoS One.

[6] Ashwood P, Krakowiak P, Hertz-Picciotto I, Hansen R, Pessah I, Van de Water J (2011). Elevated plasma cytokines in autism spectrum disorders provide evidence of immune dysfunction and are associated with impaired behavioral outcome. Brain Behav Immun.

[7] Vargas DL, Nascimbene C, Krishnan C, Zimmerman AW, Pardo CA (2005). Neuroglial activation and neuroinflammation in the brain of patients with autism. Ann Neurol.

[8] Smith ER, Nilforooshan R, Weaving G, Tabet N (2011). Plasma fetuin-A is associated with the severity of cognitive impairment in mild-to-moderate Alzheimer's disease. J Alzheimers Dis.

[9] Shi X, Ohta Y, Liu X, Shang J, Morihara R, Nakano Y (2019). Acute Anti-Inflammatory Markers ITIH4 and AHSG in Mice Brain of a Novel Alzheimer's disease Model. J Alzheimers Dis.

[10] Mori K, Emoto M, Inaba M (2011). Fetuin-A:a multifunctional protein. Recent Pat Endocr Metab Immune Drug Discov.

[11] Li W, Zhu S, Li J, Huang Y, Zhou R, Fan X (2011). A hepatic protein, fetuin-A, occupies a protective role in lethal systemic inflammation. PLoS One.

[12] Harris VK, Donelan N, Yan QJ, Clark K, Touray A, Rammal M (2013). Cerebrospinal fluid fetuin-A is a biomarker of active multiple sclerosis. Mult Scler.

[13] Puchades M, Hansson SF, Nilsson CL, Andreasen N, Blennow K, Davidsson P (2003). Proteomic studies of potential cerebrospinal fluid protein markers for Alzheimer's disease. Brain Res Mol Brain Res.

[14] Kitamura Y, Usami R, Ichihara S, Kida H, Satoh M, Tomimoto H (2017). Plasma protein profiling for potential biomarkers in the early diagnosis of Alzheimer's disease. Neurol Res.

[15] Laughlin GA, McEvoy LK, Barrett-Connor E, Daniels LB, Ix JH (2014). Fetuin-A, a new vascular biomarker of cognitive decline in older adults. Clin Endocrinol (Oxf).

[16] Fanelli G, Benedetti F, Wang SM, Lee SJ, Jun TY, Masand PS (2020). Reduced plasma Fetuin-A is a promising biomarker of depression in the elderly. Eur Arch Psychiatry Clin Neurosci.

[17] Parker W, Hornik CD, Bilbo S, Holzknecht ZE, Gentry L, Rao R (2017). The role of oxidative stress, inflammation and acetaminophen exposure from birth to early childhood in the induction of autism. J Int Med Res.

[18] Kanno T, Yasutake K, Tanaka K, Hadano S, Ikeda JE (2017). A novel function of N-linked glycoproteins, alpha-2-HS-glycoprotein and hemopexin:Implications for small molecule compound-mediated neuroprotection. PLoS One.

[19] Brettschneider J, Lehmensiek V, Mogel H, Pfeifle M, Dorst J, Hendrich C (2010). Proteome analysis reveals candidate markers of disease progression in amyotrophic lateral sclerosis (ALS). Neurosci Lett.

[20] Al-Ayadhi L,  Alhowikan AM, Halepoto DM (2018). Impact of Auditory Integrative Training on Transforming Growth Factor-β1 and Its Effect on Behavioral and Social Emotions in Children with Autism Spectrum Disorder. Med Princ Pract.

[21] Al-Ayadhi L, Halepoto DM (2013). Role of proteomics in the discovery of autism biomarkers. J Coll Physicians Surg Pak.

[22] Wang H, Li W, Zhu S, Li J, D'Amore J, Ward MF, Yang H (2010). Peripheral administration of fetuin-A attenuates early cerebral ischemic injury in rats. J Cereb Blood Flow Metab.

[23] Dziegielewska KM, Andersen NA, Saunders NR (1998). Modification of macrophage response to lipopolysaccharide by fetuin. Immunol Lett.

[24] Lane AE, Young RL, Baker AE, Angley MT (2010). Sensory processing subtypes in autism:association with adaptive behavior. J Autism Dev Disord.

[25] Schaaf RC, Toth-Cohen S, Johnson SL, Outten G, Benevides TW (2011). The everyday routines of families of children with autism:examining the impact of sensory processing difficulties on the family. Autism.

